# The Roles of Temperature-Related Post-Transcriptional Regulation in Cereal Floral Development

**DOI:** 10.3390/plants10112230

**Published:** 2021-10-20

**Authors:** Dominique Hirsz, Laura E. Dixon

**Affiliations:** School of Biological Sciences, University of Leeds, Leeds LS2 9JT, UK; bs16dsh@leeds.ac.uk

**Keywords:** cereal crops, temperature, alternative splicing, non-coding RNA, breeding targets

## Abstract

Temperature is a critical environmental signal in the regulation of plant growth and development. The temperature signal varies across a daily 24 h period, between seasons and stochastically depending on local environmental events. Extracting important information from these complex signals has led plants to evolve multiple temperature responsive regulatory mechanisms at the molecular level. In temperate cereals, we are starting to identify and understand these molecular mechanisms. In addition, we are developing an understanding of how this knowledge can be used to increase the robustness of crop yield in response to significant changes in local and global temperature patterns. To enable this, it is becoming apparent that gene regulation, regarding expression and post-transcriptional regulation, is crucial. Large transcriptomic studies are identifying global changes in spliced transcript variants and regulatory non-coding RNAs in response to seasonal and stress temperature signals in many of the cereal crops. Understanding the functions of these variants and targets of the non-coding RNAs will greatly increase how we enable the adaptation of crops. This review considers our current understanding and areas for future development.

## 1. Introduction

Temperature is an important signal in the regulation and timing of plant development. Cereal crops, including wheat, rice, and maize, which underpin the global food supply, are sensitive to temperature fluctuations. Future climate change, which is predicted to lead to global increases in temperature, means that significant crop losses are anticipated [1]. Current projections indicate that for each 1 °C increase in temperature, global yields will decrease by approximately 6% for wheat and around 10% for rice [1]. Much of this yield loss will link with alterations to the timing of reproductive development, regarding meristem transitions, flowering time, and grain filling. Plant reproductive development is particularly temperature sensitive as the mis-regulation of this causes sterility and yield loss (e.g., heat stress [2], vernalization [3], and freezing [4]). Research in crop species and the model plant *Arabidopsis thaliana* have identified a selection of temperature-responsive genes associated with these developmental stages. These include genes regulating vernalization—the seasonal requirement for a long period of cold before floral development—for example, in Arabidopsis *FLOWERING LOCUS C* (*AtFLC*) [5], *MADS AFFECTING FLOWERING 2* (*AtMAF2*) [6], *VERNALIZATION INSENSITIVE 3* (*VIN3*) [7] and in cereals *VERNALIZATION 1* (*VRN1*) ([8,9]), *VERNALIZATION 2* (*VRN2*) [10], and *ODDSOC2* (*OS2*) [11], as well as genes involved in freezing tolerance *COLD-BINDING FACTORS* (*CBFs*) ([12,13]) and heat stress *HEAT STRESS TRANSCRIPTION FACTORS (HSFs),* and *HEAT SHOCK PROTEINS (HSPs)* ([14,15]). With gene identification, it has become increasingly apparent that it is often not the presence or absence of a gene that is enabling temperature-dependent signaling, but the mechanism by which the gene regulation occurs at the post-transcriptional level. The temperature-sensitive regulation and modifications which have been identified include alternative gene splicing (AS), microRNA (miRNA), and long non-coding RNA (lncRNA) regulation.

We need to develop our molecular understanding of how plants signal in response to temperature to select optimal allelic combinations to enable the maintenance or improvement of crop yield for a wider range of environmental conditions. Understanding these post-transcriptional modifications may offer mechanisms to simultaneously regulate transcription factor families, gene clusters, and ploidy genomes in response to temperature and so offer new breeding targets in the development of temperature robust germplasm. In this review, we present our current understanding of temperature-related post-transcriptional modifications, focusing on research from temperate cereal crops and highlight the potential for these modifications in developing temperature robust germplasm for the future.

## 2. Temperature-Dependent Alternative Splicing

Alternative splicing is a post-transcriptional regulatory process which results in more than one isoform of a gene [16]. This mechanism provides an additional level of regulation in gene expression prior to protein translation, influencing the transcriptome and subsequently the proteome with variants of genes with altered functions from the same original gene copy [16]. The function of specific isoforms can vary due to a variety of modifications, such as removal of exons or retention of introns. The latter can introduce non-functional copies of gene transcripts, as retention of particular introns can introduce premature termination codons (PTC), which result in truncated, non-functional expressed gene copies. Alternative splice variants of genes exist for many plant genes, with approximately 60% of Arabidopsis genes having multiple isoforms [17]. Proportions of different alternative splice variant isoforms can reflect environmental factors such as temperature and photoperiod response, as well as intrinsic factors such as age [18]. Of particular interest for this review is temperature-dependent alternative splicing, which is a thermosensing mechanism that is sensitive to even minor changes in ambient temperature [19]. This mechanism is involved in regulating key developmental processes, including the transition from vegetative to floral development, circadian clock entrainment, and temperature-compensation of circadian period [20,21,22]. The effect of temperature-dependent alternative splicing on these areas will be described further, with a particular focus on crop species where temperature-dependent alternative splicing has been identified.

### 2.1. Alternative Splicing in the Circadian Clock

**Figure 1:**
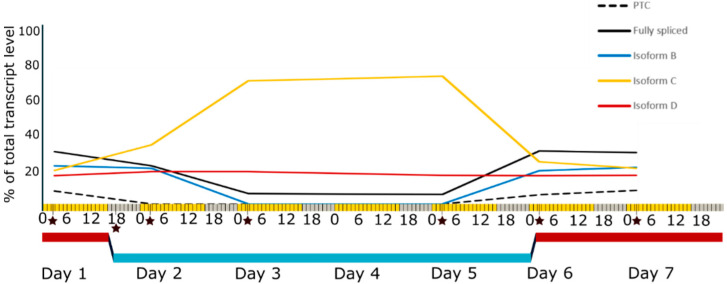
Expression of barley *Ppd-H1* isoforms shows variation in exon 6 splicing at different temperatures. The relative expression of different isoforms in exon 6 of *Ppd-H1* measured at different time-points, relative to total expression of *Ppd-H1* at 20 °C for expression spanning the region between exons 5 and 7. The PTC (premature termination codon) group, black dashed line, is made up of multiple transcript groups containing a premature termination codon. Transcript levels of other protein-coding isoforms showing variations in exon 6, Isoform B (blue line), Isoform C (yellow line), and Isoform D (red line) are shown. Isoform B lacks 6 nt at the 3′ end of the transcript, C lacks 45 nt at the 5′ end, and D has both alterations. The photoperiod conditions are on the x-axis, 16 h light (yellow) and 8 h dark (grey), with hours relative to dawn (0 h). Black stars indicate sampling points, which were taken 2.5 h after dawn and directly following changes in temperature, where expression was measured. Underneath the temperature for each day is indicated, 20 °C (red) for day 1 until dusk, where the temperature decreased to 4 °C (blue). This increased back to 20 °C at dawn of the sixth consecutive day and remained at this temperature for an additional day.

An accurate circadian clock is integral to ensure core biological processes, from photosynthesis to flowering, are timed optimally with the external environment. Clock genes have been identified to influence many agricultural traits, including yield potential and flowering time; thus, understanding the effect of environmental factors on the expression of these genes is vital [23]. The expression of core clock genes enables the formation of interlocking feedback loops and the maintenance of an oscillating day–night cycle across a 24 h period. Temperature-dependent alternative splicing is a key mechanism which modulates expression of many of these genes, and has been identified in multiple plant species, including Arabidopsis, barley (*Hordeum vulgare*), and sugarcane (*Saccharum officinarum*) [17,22,24]. Several genes associated with maintaining the spliceosomal activity of circadian clock genes have been identified in Arabidopsis [22]. Mutants in these genes lengthen or shorten the clocks period by regulation of alternative splicing of clock genes, with several affected by changes in ambient temperature [25,26,27]. The spliceosomal factors GEMIN2, SNW/Ski-interacting protein (SKIP) and SICKLE (SIC) are all regulators of alternative splicing in Arabidopsis, necessary for modulation of temperature inputs into the clock. The nuclear protein SIC is necessary for a correct temperature response for the clock gene *PSEUDO-RESPONSE REGULATOR 7 (PRR7),* in particular in affecting the cold ambient temperature response [25]. Alternative splicing of *PRR7* and *PSEUDO-RESPONSE REGULATOR 9 (PRR9)* is also regulated by the splicing factor SKIP, a temperature-sensitive spliceosomal component which effects multiple regulatory genes in the clock, stress tolerance, and flowering time pathways [28,29]. In cereals, the primary orthologue of the *PRR* genes is *PHOTOPERIOD-1 (Ppd-1),* which has been shown to undergo high levels of temperature-dependent alternative splicing in barley (*Hordeum vulgare*)*. Ppd-1* plays a critical role in photoperiod monitoring and response, with a promoter mutation resulting in overexpression of *Ppd-1* able to confer photoperiod-insensitivity [30]. This trait has been utilised in agriculture to generate cereal crops able to flower irrespective of daylength, which is vital for the global cultivation of naturally long-day flowering crops such as barley and wheat *(Triticum aestivum)*. *Ppd-1* in barley has multiple conserved isoforms affecting exon 6, which are expressed differently with rapid isoform switching in response to increases or decreases in temperature (Figure 1) [17]. Larger alternative splicing events occurred particularly at lower temperatures, with retention of intron 3 or skipping of exon 4 resulting in in-frame protein isoforms, though the function is unknown as no known domains are effected [17]. Furthermore, the barley *Ppd-1* gene is predicted to have a total of 40 alternative splicing variants and so highlights the possibility of a vast array of transcript regulation in response to environmental factors. In hexaploid wheat *(Triticum aestivum)*, the primarily expressed *Ppd-1* genome copy on the D genome has seven predicted alternative splicing isoforms, which is suggestive of regulation similar to that identified in barley regarding temperature-dependent alternative splicing.

A further spliceosomal assembly factor, GEMIN2, also alters the splicing of core clock genes including *TIMING OF CAB EXPRESSION 1 (TOC1)* under cold temperatures in Arabidopsis, acting as a buffer to modulate the effects of cold conditions on alternative splicing [26]. The circadian clock gene *CIRCADIAN CLOCK-ASSOCIATED1 (CCA1)* is also known to have a role in cold stress, with the *CCA1α* isoform involved in regulating genes enabling freezing-tolerance whilst the isoform *CCA1β* integrates temperature signals into the clock and competes with *CCA1α* [31]. This offers an interesting mechanism which could potentially be used in the development of frost-tolerant crops.

Temperature-dependent alternative splicing of the core clock genes is also vital in regulating the circadian clock of sugarcane (*Saccharum* hybrid). Here, it effects several *ScPRR* genes as well as *ScTOC1* and *LATE ELONGATED HYPOCOTYL (ScLHY)* to regulate the abundance of these genes transcripts across seasons [24]. In particular, *ScLHY* is alternatively spliced to produce an abundance of non-productive isoforms at lower temperatures [24]. In barley, an increase in alternative splicing of *HvLHY* has been identified at low temperatures, as well as an overall decrease in expression [17].

Further improving our understanding regarding how temperature-dependent alternative splicing is regulating the cereal circadian clock and the subsequent impact on traits will be important to ensure the adaptation of crops under variable temperature conditions. It is particularly fascinating if different transcript isoforms are used to regulate distinct pathways in floral regulation and development. Targeting genes which undergo temperature dependent alternative splicing in these pathways could allow adaptation to specific traits, such as meristem development or stem elongation, without impacting the core circadian mechanism.

### 2.2. Alternative Splicing and the Floral Transition

Floral identity and transition is regulated in part by several MADS-box transcription factors, which modulate the expression of the well-conserved central floral signal integrator, *FLOWERING LOCUS T (FT)*. *FT* is present in all angiosperms and largely functions as the predicted ‘florigen’. The MADS-box transcription factors involved in the regulation of *FT* vary between plant species and these are influenced by different environmental factors, such as temperature and photoperiod. This regulation optimises the timing of floral transition to minimise environmental damage to the highly sensitive floral organs. Alternative splicing of *FT* has been identified as responsible for the earlier flowering of the dwarf variety of coconut following regulation by genes involved in the photoperiodic pathway, including *TOC1* [32]. Alternative splicing of *FLOWERING LOCUS T–like 2 (FT2)* has also been identified as age-dependent in the model grass *Brachypodium distachyon,* and both *FT2* and the alternative spliced isoforms are conserved in wheat and barley [18]. The two isoforms *BdFT2α* and *BdFT2β* have notably different effects on floral transition. *BdFT2β* acts as a dominant negative regulator as it is unable to form complexes with *FD* whilst still interacting with *FT1* and *FT2α* to prevent these from promoting flowering [18]. Identifying if the alternative splicing regulation of cereal *FTs* is also temperature-regulated would be particularly important for identifying selective breeding targets.

In Arabidopsis, there are a number of key floral regulators which undergo temperature-dependent alternative splicing, including members of the *MADS AFFECTING FLOWERING (MAF)* gene clade, which contains the floral repressor *FLC* [33]. This family includes *FLOWERING LOCUS M (FLM)*, alternatively called *MAF1*, as well as *MAF2,* which are floral repressors and so are down-regulated to enable floral transition [6,21]. *FLM* acts as a thermosensor and forms a complex with another MADS-box floral repressor, SHORT VEGETATIVE PHASE (SVP), to suppress *FT* expression [21,34]. Loss of function *flm* mutants are partially temperature-insensitive and flower early, whilst constitutive overexpression results in a late flowering phenotype [21,35]. The two major isoforms of *FLM* are *FLM-β* and *FLM-δ*, with alternative use of exons 2 and 3, respectively [36]. These two isoforms compete to interact with SVP at different ambient temperatures as a regulatory mechanism. At cooler temperatures, *FLM-β* forms a functional heterodimer with SVP to repress flowering, whilst warmer temperatures result in *FLM-δ* preferentially forming a complex [34]. The latter isoform is non-functional and therefore unable to bind with the *FT* gene to regulate expression. Through this competitive interaction mechanism between the two isoforms, *FLM-δ* outcompetes the repressive function of the *SVP:FLM-β* complex when the ratio of *FLM-β:FLM-δ* isoforms has been sufficiently altered. Alternative splicing of *FLM* is mediated by the splicing factor *SF1*, which is particularly required to generate the *FLM-δ* isoform at warmer temperatures [37]. This splicing regulation is via recognition of the 3′ splice site and binding of *SF1* to the intron branch point. Mutations in the RNA recognition motif (RRM) domain primarily alter production of the *FLM-β* isoform, and therefore, subsequently alter flowering time [38]. In addition to *SF1*, temperature-dependent alternative splicing of *FLM* is regulated by a complex made up of the cyclin-dependent kinase G2 (CDKG2) and CYCLYN1 (CYCL1) [39]. This complex specifically regulates splicing of introns 1 and 4 of *FLM*, with reduced intron 4 retention in *cdkg2/cycl1* mutants. These double mutants are early flowering, indicating that the activity of the *CDKG2/CYCL1* complex is necessary to generate the *FLM* isoforms required for suppressor activity [39].

*MAF2* is another member of the same gene family that acts in tandem with *FLM,* also undergoing temperature-dependent alternative splicing and interacting with SVP but via a different mechanism to *FLM* [6]. As with *FLM, MAF2* generates an isoform predominant at cooler temperatures (*MAF2 var1*), which forms a functional heterodimer with SVP to repress flowering. In warmer ambient temperatures, intron 3 of *MAF2* is retained, which introduces an in-frame PTC to generate *MAF2 var2*. This results in a truncated isoform, which lacks the entire C-domain and part of the K-domain, needed for dimer formation to form a complex with SVP [6]. Therefore, the *MAF2 var2* isoform is not functional and unable to repress the floral transition [6]. *MAF2* splice variants 1 and 2 are both highly expressed and conserved in crops *Brassica rapa* and *Brassica napus,* suggesting conservation of alternative splicing in *MAF2* function [33]. *MAF2* also has a role in preventing vernalization by short periods of cold in Arabidopsis, acting as a floral repressor in a pathway independent of *FLC* [40]. This suggests that this family of floral repressors regulate temperature response through similar yet distinct mechanisms, to regulate floral transition. Whilst there are no direct homologs of the *MAF* family in the cereals, there are many MADS-box genes and the temperature-dependent alternative splicing mechanisms described in Arabidopsis are likely to be present. However, due to the additional regulation though copy number variation and genome-specific activity found in many of the polyploid crop species, we can anticipate identifying a more complex regulatory network in the temperature-sensing and temperature-responsive genes.

## 3. Temperature-Responsive Non-Coding RNAs

Non-coding RNAs are involved in transcript regulation in various types of temperature responses, including the slower developmental response of vernalization through to the rapid high temperature and freezing stress responses. Of the many classes of non-coding RNAs, there are two important regulatory groups involved in temperature responses: the 20–22 nucleotide micro RNA (miRNA), and the long non-coding RNA (lncRNA) of variable lengths (usually >200 bp). Transcriptome studies have identified dramatic changes in the levels of non-coding RNAs in response to temperature changes. For example, in wheat stress signaling responses, e.g., to high or low temperatures and to drought, which often have the consequence of accelerating or aborting floral development, large increases in non-coding RNA expression have been reported ([41,42]). One of the major outstanding challenges is to identify the targets of these non-coding RNAs and further understand the function in temperature signaling.

### 3.1. Temperature-Responsive miRNA

MiRNAs are transcribed by RNA polymerase II (pol II) into longer pre-miRNAs which, in this intermediate state, form self-complementary hairpin loop structures. These are subsequently cleaved via DICER-like 1 endonucleases and processed before the miRNA is bound to ARGONAUTE 1 protein and used as a guide to identify complementary transcripts and target these for degradation. In hexaploid wheat (*Triticum aestivum*), one of the most important genes involved with the domestication of the crop, *Q*, is regulated by miR172 [43]. A single nucleotide polymorphism (SNP) in the miR172 binding site in *Q* alters transcript level causing an elongation in the spike (the wheat flower) shape and alteration in how brittle the spike is. This was essential for the processing of the wheat spike as a harvestable crop [43]. The importance of miRNAs in wheat and other polyploid crops may reflect the requirement, following the hybridization events, to simultaneously control multiple, highly similar genomes. Additionally, the multiple targets of a single miRNA also provides a mechanism to simultaneously regulate and integrate distinct signaling pathways. For example, miR319 targets a number of the members of *TEOSINTE BRANCHED 1, CYCLOIDEA, PROLIFERATING CELL FACTOR 1* (*TCP)* transcription factor family to coordinate the biosynthesis of jasmonic acid. During heat stress, miR319 enables the simultaneous downregulation of the *TCP* targets, thus coordinating stress responses with hormone signaling and development, via the TCPs [44]. The TCPs, in particular *TEOSINTE BRANCHED 1*, are known to be important in the regulation of cereal plant and spike architecture and so offer an interesting potential target for miR319 regulation in response to temperature signals [45].

**Figure 2:**
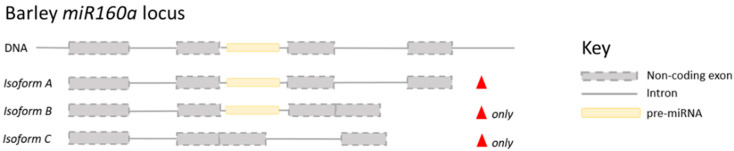
The lncRNA isoforms which contain the barley miR160a. All of the isoforms show increases in expression following heat shock (denoted by the red triangle). *Isoform A* is the full lncRNA, *Isoform B* has had intron 3 spliced, and *Isoform C* has had intron 2, which contains pre-miR160a, spliced. *Isoforms B* and *C* are only found following heat stress, denoted by the red triangle followed by only [47]. The image is not to scale.

Transcriptome studies have provided an excellent insight into the global abundance changes of miRNAs in response to certain environmental signals. Significant changes in miRNA abundance were identified via RNA-seq analysis in response to heat stress in wheat [41], maize [46], barley [47], and to freezing stress [48]. However, only relatively few miRNAs have been characterised to the level of target identification in response to temperature signals. In hexaploid wheat, miR159 was identified to be significantly upregulated following heat stress [49]. Target identification showed that miR159 was regulating the transcription factor *GAMYB1* and the downregulation of miR159 enabled an upregulation of *GAMYB1* [50]. Conversely plants constitutively expressing miR159 from the ubiquitin promoter withered more and grew more slowly following heat stress [50]. Much of the characterization of miR159 responses in wheat has been conducted in more readily transformable plant species, including rice and Arabidopsis, and highlights the conserved nature of many miRNAs. However, similar to genes, whilst miRNAs are well conserved between species, the critical basepair or regulatory factor is species or even cultivar specific. An interesting additional layer of regulation has been identified in barley where four miRNAs (miR160a, 166a, 167h and 5175a) were identified to be up-regulated in response to heat stress but for miR160a and 5175a, the introns within which the miRNAs are located were also spliced in a heat stress regulated manner [47]. For example, miR160a was identified to be encoded within a non-coding transcript, which contained 3 introns, following a 6 h heat stress treatment. Two additional spliced isoforms (*Isoforms B* and *C*) of this transcript were identified, which were not found in non-heat stressed plants (Figure 2) [47]. *Isoform C* was shown to lack intron 2 which codes for the pre-miR160a (Figure 2). The targets of the heat stress responsive miRNAs include *HD-Zip transcription factors*, *AUXIN RESPONSE FACTORS* (*ARFs*), *HOMEOBOX-LEUCINE ZIPPER PROTEIN HOX9* *(HOX9)*, *1-AMINOCYCLOPROPANE-1-CARBOXYLIC ACID OXIDASE* *(ACC oxidase)* and *Nek5-like kinase,* which were identified to be down-regulated in response to heat stress. A number of these genes link with floral development and architecture. The large number of gene targets, combined with the double layer of miRNA high-temperature mediated regulation, indicates the importance of miRNAs within this response.

Temperature-mediated miRNA regulation is not only linked with high temperatures; the role of miRNAs has also been determined in low ambient and vernalizing temperatures. In *Brachypodium distachyon,* miR156 has been identified to regulate gene expression at lower ambient temperatures. The target of miR156 is the *VERNALIZATION INSENSITIVE 3-like* (*VIN3-like)* gene *BdVIL4,* as determined through transgenic studies which showed that *BdVIL4* RNAi plants phenocopied *miR156-ox* plants [51]. The analysis suggested that, similar to Arabidopsis, miR156 and *VIL4* co-regulated each other to regulate and modulate the developmental response to ambient low temperatures [51]. In wheat, developing spikes exposed to cold stress resulted in 39 differentially expressed miRNA between cold stress and control plants [48]. A number of the miRNAs were predicted to regulate floral organ identity genes, including *ARFs*(*AUXIN RESPONSE FACTORS*), *SPB* (*SQUAMOSA PROMOTER BINDING-like*), and numerous examples from the transcription families of MADS-box and *TCP* genes [48]. Such a large number of genes being regulated by cold-stress induced miRNA expression suggests that the development is extremely cold temperature sensitive and that floral architecture as well as timing may be regulated by cold-stress events. Again, further identification of miRNA targets and how these are regulated under variable temperatures will facilitate the rapid adaptation of these developmental traits.

### 3.2. Temperature-Responsive LncRNA

The second major class of temperature-responsive non-coding RNA are the long non-coding RNAs (lncRNAs). LncRNAs are often but not exclusively expressed from the anti-sense of a gene and are a complementary copy of part of the transcribed mRNA. Usually there is not a single, specific lncRNA but a population of highly similar transcripts of varying lengths. These transcripts, like genes, are poly-adenylated, can contain introgenic regions, and can therefore be regulated through splicing mechanisms. What lncRNAs lack is the potential to code for protein product (reviewed by [52]). Evidence from vernalization regulation in Arabidopsis, regarding the *COOLAIR* transcripts associated with the MADS-box transcription factor *FLOWERING LOCUS C* (*AtFLC*) show that some of these different lncRNA alleles have unique functions regarding the regulation of gene expression in response to environmental conditions [53]. Similar to miRNAs, the lncRNAs can function through the formation and targeting of dsRNA for degradation; however, they can also regulate transcription via many other molecular methods, some of which are described below.

**Figure 3:**
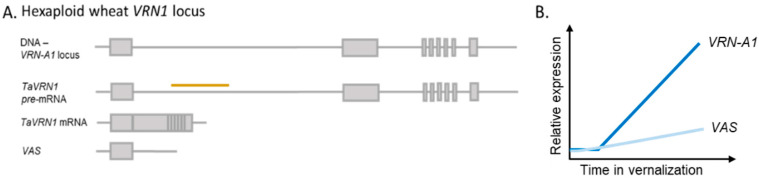
The known isoforms from *TaVRN1* locus. (**A**) The *VRN-A1* locus within the hexaploid wheat genome and the expressed transcripts (pre-mRNA and *VAS*) as well as the spliced mRNA. The yellow line indicating the position of the regulatory motif in intron 1 (**B**) A schematic of the expression of *VRN-A1* and *VAS* during vernalization.

As a regulatory class of ncRNA comparatively less is known about lncRNA, however global changes in ncRNA identify strong differential expression under different temperatures. The function of ncRNA as distinct temperature mediated regulators is highlighted by the characterization of a group of lncRNAs in Arabidopsis that are involved in the regulation of the vernalization gene *FLOWERING LOCUS C* (*FLC*) transcript abundance. In the absence of warm temperatures, a population of lncRNAs are transcribed from the antisense strand at the 5′ end of *FLC*, termed *COOLAIR* [54]. These lncRNAs have been characterized into three sub-groups which differentially act to target the *FLC* transcript for degradation. Whilst there are variable reports of the requirement for cool/vernalizing temperatures to trigger their expression, recent work has identified the role of freezing temperatures to initiate *COOLAIR* transcript, indicating that the first frost event of autumn is important in initiating the vernalization process in the field [55]. In cereals, there are relatively few gene-associated lncRNAs identified; however, one interesting temperature related example shows homology with the *COOLAIR* regulation of *AtFLC*. In monocots, including rice, maize, wheat, and Brachypodium, lncRNA transcripts have been associated with a temperature-regulated MADS-box gene *ODDSOC2* (*OS2*) although whether they function in vernalization remains to be determined [56]. Expression of relatively well conserved, compared to other flanking regions, lncRNA occurs from the antisense strand of *BdODDSOC1* and *BdODDSOC2.* Knock-downs of *BdCOOLAIR2* transcripts did not generate any morphological phenotype but they did result in an increase in the abundance of the *BdODDSOC2* transcript [56]. This suggests that a similar molecular function exists between the dicot and monocot versions of this gene and that perhaps in species with a more pronounced vernalization response the mechanism may have morphological importance. A recent publication has also identified a lncRNA *TaVRN1 alternative splicing* (*VAS*) that is alternatively spliced from the sense strand of the central cereal vernalization gene *VERNALIZATION 1* (*VRN1*) (Figure 3) [57]. Interestingly, rather than target *VRN1* transcript for degradation, *VAS* actually recruits bZIP transcriptional factors (TaRF2b that binds TaRF2a), which increase the expression of *VRN1*. Through the expression of *VAS* in the early stages of vernalization, additional *VRN1* expression is observed in the mid-vernalization stage and so accelerates the time to flowering [57]. Further understanding the genome specificity of *VAS* expression, the role of copy number variation at the *VRN1* locus, along with understanding its regulation under field conditions are areas of particular interest.

The role of lncRNAs as regulators in low-temperature signaling extends to freezing temperatures. A genomic study of a Chinese cultivar (Dongnongdongmai 1) identified 7435 lncRNAs that showed differential expression in response to cold temperatures and were co-expressed with 7191 mRNAs [58]. Furthermore the study identified that a potential 621 mRNAs were being regulated through potential competitive regulation of lncRNA and miRNA, highlighting an interesting intersection for these two pathways [58]. Low/freezing temperature regulation by lncRNAs is also observed in Arabidopsis through the expression regulation of *COLD-BINDING FACTOR 1* (*CBF1)* by the *lncRNA SVALKA* (*SVK*) [59], again identifying an interesting regulatory mechanism which may exist in cereals, as the *CBF* genes are well conserved. An increase in the number of lncRNAs is also observed in durum wheat, with 31 lncRNAs being differentially expressed between cold (5 °C) and control conditions in the spring durum cultivar CBW0101 [60].

## 4. Protein Modifications Linking with Post-Transcriptional Regulation

*VRN1* is the major, dominant regulator of vernalization in cereals and has therefore been the subconscious target for adaptive selection for the regulation of vernalization. This selection has enabled the subsequent identification of an important regulatory region within intron 1 of *VRN1* in hexaploid wheat [61]. A deletion of a section of the *VRN1* intron 1 results in a more constitutive expression of *VRN1* and so confers spring growth habit [61]. *VRN1* is a MADs-box transcription factor, which like *FLC* is temperature and post-transcriptionally regulated, as mentioned above. A mechanism involving post-transcriptional and protein modification regulation has been identified for the intron 1 region of *VRN1*. The pre-mRNA of *VRN1* is bound by the glycine-rich RNA binding protein 2 (TaGRP2) via a RIP3 motif and this limits *VRN1* transcript accumulation and therefore prevents vernalization from proceeding. During the low temperatures associated with vernalization, a carbohydrate-binding protein *VERNALIZATION RELATED GENE 2* (VER2) interacts with GRP2, an interaction which is enhanced through O-GlcNAcylation (O-GlcNAc) of GRP2. These interactions reduce the amount of GRP2 binding *VRN1* pre-mRNA and so allows the accumulation of *VRN1* transcript and therefore protein [62]. Further analysis has identified that GRP2 is dynamically regulated by both O-GlcNAc and phosphorylation during vernalization, indicating a possible regulatory mechanism for determining the duration of cold during vernalization [63]. Interestingly the study also identified 31 vernalization-associated proteins that are modified by both O-GlcNAc and phosphorylation modifications [63]. This suggests that protein modification, combined with post-transcriptional regulation is important as part of the mechanism by which cereals measure temperature fluctuations.

## 5. Concluding Remarks

Utilizing how crop plants integrate environmental information has always been important to enable optimal cultivation. However, with the rapid environmental changes occurring under climate change, the importance of optimal adaptation of crops is rising in prominence again. To enable rapid adaption, we will need to utilise all of the possible regulatory mechanisms. The research reviewed in this manuscript highlights some of the possibilities for targeted temperature-regulated adaptation in crops through post-transcriptional modifications. Identifying breeding targets to preferential selection for specific isoforms of genes or alter binding affinities of ncRNAs may offer temperature-specific regulatory mechanisms. Many more molecular mechanisms could be temperature-regulated, such as organelle storage of transcripts, and so offers an exciting future for crop research.

## Data Availability

Not applicable.
